# Myocarditis Caused by *Brucella melitensis* in the Absence of Endocarditis: Case Report and Review of the Literature

**DOI:** 10.1155/2019/3701016

**Published:** 2019-02-10

**Authors:** Maria Lagadinou, Virginia Mplani, Dimitrios Velissaris, Periklis Davlouros, Markos Marangos

**Affiliations:** ^1^Internal Medicine Department, University Hospital of Patras, Rio, Greece; ^2^Emergency Department, University Hospital of Patras, Rio, Greece; ^3^Department of Infectious Diseases, University Hospital of Patras, Rio, Greece; ^4^Cardiology Department, University Hospital of Patras, Rio, Greece

## Abstract

Brucellosis remains an important public health problem with endemic characteristics in many countries. Brucellosis can affect almost all organs and systems of human body. Cardiac complications are unusual, occurring in less than 2% of patients and usually manifest as endocarditis. We present the case of a 21-year-old Caucasian man, who was admitted to the University Hospital of Patras, Western Greece, with fatigue, fever up to 39°C, and retrosternal pain. Musculoskeletal, genitourinary, gastrointestinal, hematologic, nervous, skin, and mucous membranes and respiratory complications have been reported in several cases of brucellosis. Development of myocarditis is a highly rare complication of brucellosis, particularly in the absence of concomitant endocarditis. Clinicians should be aware of this clinical entity especially in endemic areas as appropriate antibiotic treatment is life-saving and may prevent serious cardiologic disorders.

## 1. Introduction

Brucellosis remains an important public health problem with endemic characteristics in many countries [1]. It is a worldwide zoonosis, with Mediterranean basin, the Middle East, India, Mexico, and Central and South America being the areas with the highest prevalence [2]. Brucellosis can affect almost all organs and systems of human body and should always be considered in the differential diagnosis in patients presenting with chronic fever combined with a history of contact with animals or animal products [3].

Cardiac complications from brucellosis are unusual, occurring in less than 2% of patients and usually manifest as endocarditis [4]. Unlike endocarditis, which is the most common cardiac complication, acute pericarditis and myocarditis without associated endocardial involvement are rare, and very few cases have been reported from countries with high prevalence of the disease [5]. Herein, we present an unusual case of a patient with myocarditis due to *Brucella* in the absence of concomitant endocarditis.

## 2. Case Presentation

A 21-year-old Caucasian man was admitted to the University Hospital of Patras, Western Greece, with fatigue, fever up to 39°C, and retrosternal pain. He denied anorexia, night sweats, and generalized malaise. No significant past medical history was reported. There were no risk factors for HIV infection, no recent travel outside Greece, and no exposure to animals. The patient denied smoking and drinking, and also no allergies were noted.

On physical examination, the temperature was 39.0°C, the heart rate was 90°bpm with sinus rhythm, and the blood pressure was 120/80 mmHg. The patient was respiratory stable (respiratory rate 16/min and oxygen saturation 98% on room air). No cervical or supraclavicular lymphadenopathy was identified. There were no murmurs, rubs, or gallops, and the lungs were clear on auscultation and percussion. The abdomen was nondistended, with normal active bowel sounds and mildly tender in the midepigastrium but without rebound or guarding. No liver or spleen enlargement was noted. No abnormalities like clubbing, cyanosis, or edema were found on all extremities. The rest of the examination was unremarkable.

**Figure 1:**
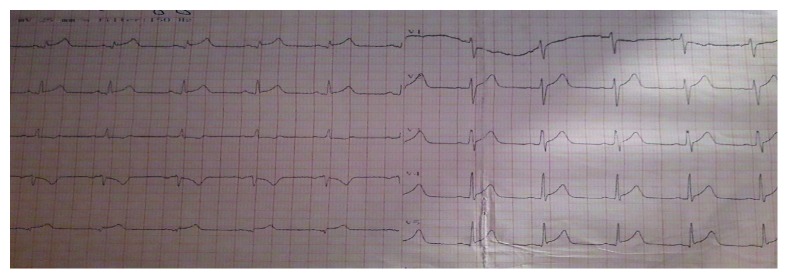
ECG findings upon admission: sinus rhythm with ST elevation (ST 2 mm in I, II, aVL, and V4–V6).

**Figure 2:**
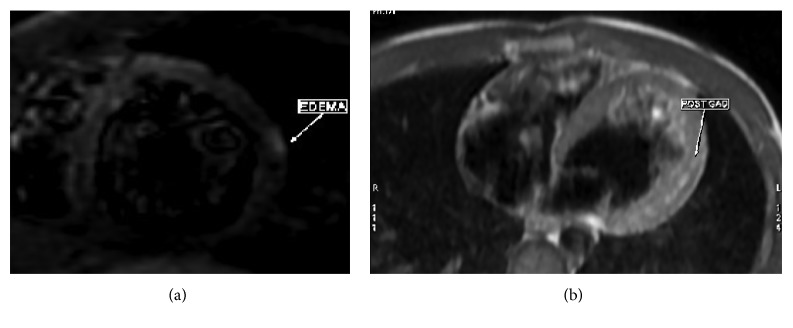
T2 STIR sequence, showing localized high intensity signal on the lateral midepicardial wall of the LV, corresponding to inflammation edema. Hyperemic post-Gad sequence: high intensity signal in the midmyocardial segment of the midlateral LV wall, corresponding to inflammation and/or fibrosis.

**Figure 3:**
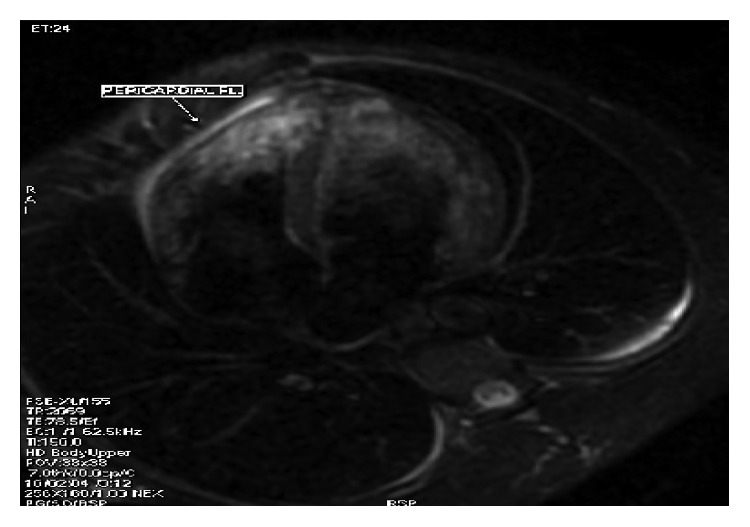
Small bilateral pleural effusion and localized pericardial effusion around the anterolateral RV wall.

Electocardiography (ECG) revealed a sinus rhythm with ST elevation (ST 2 mm in I, II, aVL, and V4–V6) (Figure 1). Furthermore, laboratory tests showed a low platelet count 134.000 (normal range 150.000–400.000 Κ/*μ*l), raised aspartate aminotransferase (193 U/L, upper normal limit (UNL) 40 U/L) and alaninoaminotranferase (42 U/L 40 U/L), high CPK levels (2166 mg/dl, upper normal limit 190 mg/dl) with CPK-ΜΒ lower than 10% of total CPK (112 mg/dl), troponine (ΤnI) 48.51 ng/ml, and CRP 4.60 mg/dl. The hemogram was normal (Table 1). Chest X-ray image did not reveal any abnormalities. Blood and urine cultures were taken on admission. The transthoracic echocardiography Doppler showed wall motion abnormalities and absence of pericardial effusion. Accordingly, a cardiac MRI using delayed enhancement was performed (Figures 2 and 3) revealing recent myocardial damage with edema and fibrosis in the middle and upper left and right lower wall and increased left ventricular dimensions with normal systolic function.  In addition, cardiac MRI revealed overriding of the right ventricle with normal systolic function

Serology for *Influenza A* and *B*, parvovirus B19, EBV, and CMV, ECHO virus, *Coxsackie* virus, HSV, VSV, and adenovirus, *Coxiella burnetii*, *Chlamydia*, *Leptospira* spp., and *Mycoplasma pneumonia*e were negative. On day 3 of hospitalization, *Brucella melitensis* was isolated from two consecutive blood cultures. The *Brucella* serum agglutination test (SAT) was positive >1/1280, so a diagnosis of *Brucella*-related myocarditis was made. Treatment with oral rifampicin (900 mg once daily) and doxycycline (100 mg twice daily) along with intravenous gentamycin (320 mg once daily) was immediately commenced. Gentamycin was administered for ten days. The patient recalled that he had consumed unpasteurized goat cheese a month ago. After five days of treatment, the patient was clinically improved, asymptomatic, and fever was regressed. No signs of cardiac arrythmias or other ECG abnormalities on serial ECGs, during hospitalization, were noted. On discharge day, all laboratory tests were normal (Table 2). Patient had a total antibiotic course with Doxycycline and rifampicin for 6 months.

## 3. Discussion

Brucellosis is a zoonosis infecting the human, having a worldwide distribution especially in the developing countries [6]. The microorganism is frequently transmitted to humans via consumption of infected unpasteurized dairy products and direct contact with infected animal tissues. The prevalence of the disease is high in the Arabian Peninsula and Mediterranean countries [4]. Clinical presentations of brucellosis are various. The most common symptoms of the disease are fever (95%), anorexia (90%), fatigue (90%), smelly perspiration (80%), arthralgia (25–50%), and weight loss. Less common symptoms and signs of the disease are swelling of the joints (15%), splenomegaly (20%), and lymphadenopathy of the inguinal area (10–15%). Bronchitis, pleurisy, emphysema, pulmonary abscess, and cardiac involvement are very uncommon [5].

Infection from *Brucella* species has a wide range of clinical complications. Musculoskeletal, genitourinary, gastrointestinal, hematologic, nervous, skin, and mucous membranes and respiratory complications have been reported in several cases. Cardiovascular involvement is a rare complication and usually is presented as endocarditis, remaining the principal cause of mortality in the course of brucellosis. It usually affects the aortic valve and typically requires immediate surgical valve replacement [4].

However, endocarditis is the most common cardiac complication of the disease. A few case reports have been published, illustrating different forms of Brucella endocarditis. In the absence of concomitant *Brucella*-related endocarditis, development of myocarditis is extremely rare. According to Colmenero et al., only 1.54% of 530 brucellosis cases had cardiac involvement, with only one patient having myocarditis [7]. Cases of pericarditis or myocarditis without simultaneous endocarditis are reported sporadically [6, 8, 9]. PubMed database search for articles published until October 2018 using keywords myocarditis and Brucella revealed only a few reports with myocarditis in the absence of endocarditis (Table 3).

In the current case report, no involvement of cardiac valves was observed in repeated echocardiography. The diagnosis of Brucella myocarditis was based on with positive blood cultures, positive Brucella serum agglutination test, and pericardial effusion in echocardiography, associated with typical symptoms and myocardial involvement as well. The mechanism of cardiac damage is not clear, but it may be due to the direct effect of the microorganism or local deposit of immunocomplexes.

Patients suffering from Brucella myocarditis usually respond to antibiotic therapy well. According to previous reports, streptomycin (1 g/day for 3 weeks) and doxycycline (200 mg/day for 6 weeks) or rifampicin (600 mg/day for 6 weeks) and doxycycline (200 mg/day for 6 weeks) are the appropriate therapy regimens [4]. Our patient was successfully treated with oral rifampicin (900 mg once daily) and doxycycline (100 mg twice daily) along with intravenous gentamycin (320 mg once a day). Gentamycin was administered for 10 days totally, while doxycycline and rifampicin were given for 6 months. This prolonged administration was decided after consultation of the Hospital's Infectious Diseases Consultation team due to the severity of the disease and the myocardial involvement. Furthermore, no clear guidelines related to this complication of brucellosis exist.

In conclusion, development of myocarditis is a highly rare complication of brucellosis, particularly in the absence of concomitant endocarditis. Clinicians should be aware of this clinical entity especially in endemic areas as appropriate antibiotic treatment is life-saving and may prevent serious cardiologic disorders.

## Figures and Tables

**Table 1:** Laboratory tests on admission.

Variable (unit)	Patient result	Reference value
Hemoglobin (g/dl)	13.9	12–15
Platelets (Κ/*μ*L)	134.000	150.000–400.000
Leukocytes (K/Μl)	4.82	4.0–11
SGOT (U/l)	193	<40
SGPT (U/l)	42	<40
CPK/CPK-ΜΒ (U/l)	2166/112	<140
ΤnI (ng/ml)	48.51	<14

**Table 2:** Laboratory findings on the discharge day.

Variable (unit)	Patient result	Reference value
Hemoglobin (g/dl)	14	12–15
Platelets (Κ/*μ*L)	184.000	150.000–400.000
Leukocytes (K/Μl)	4.85	4.0–11
SGOT (U/l)	58	<40
SGPT (U/l)	34	<40
CPK/ΜΒ (U/l)	109/10	<140
ΤnI (ng/ml)	—	<14

**Table 3:** Published case reports referring to Brucella myocarditis.

Publication	Gender	Age	Treatment	Follow-up
Gur et al. [10]	Woman	25 y	Streptomycin and tetracycline	Relapse after 4 months
Lubani et al. [11]	Boy	10 y	Tetracycline for two weeks Streptomycin for three weeks	Two-year follow-up showed no relapse
Jubber et al. [12]	Man	55 y	Doxycycline and rifampicin	No follow-up
Efe et al. [4]	Woman	51 y	Streptomycin for 3 weeks Doxycycline for 6 weeks	3-month follow-up: asymptomatic
Elkiran et al. [13]	Girl	3 months	Gentamycin, Bactrim, and rifampicin	Four-month follow-up: no relapse
Pandit et al. [6]	Woman	32 y	Streptomycin and doxycycline	Worsened and died due to pulmonary odema
Gatselis et al. [2]	Man	34 y	Streptomycin for 3 weeks Doxycycline and rifampicin	One-year follow-up: no symptoms, no relapse
Gatselis et al. [2]	Man	17 y	Streptomycin for 3 weeks Doxycycline and rifampicin	One-year follow-up: no symptoms, no relapse
Adid et al. [8]	Man	32 y	Streptomycin for 2 weeks	3-month follow-up: no relapse
			Doxycycline and rifampicin for 12 weeks	
Abid et al. 2012 [8]	Man	20 y	Cotrimoxazole and rifampicin	No follow-up
Khorasani and Farrokhnia 2014 [5]	Man	22 y	Cotrimoxazole, doxycycline, and rifampicin for 3 months	Two-month follow-up: asymptomatic
Pandit et al. 2010 [6]	Man	27 y	Doxycycline, rifampicin for 12 weeks, and gentamycin for 10 days	After several months, the patient was asymptomatic
